# Step-by-step assembly and testing of a low-cost bioprinting solution for research and educational purposes

**DOI:** 10.1016/j.mex.2020.101186

**Published:** 2020-12-17

**Authors:** John Robert Honiball, Michael Sean Pepper, Earl Prinsloo

**Affiliations:** a Biotechnology Innovation Centre, Faculty of Science, Rhodes University, Grahamstown, South Africa; b Department of Immunology, Institute for Cellular and Molecular Medicine, University of Pretoria, Pretoria, South Africa; c SAMRC Extramural Unit for Stem Cell Research and Therapy, Faculty of Health Science, University of Pretoria, Pretoria, South Africa

**Keywords:** Bioprinting, Sodium alginate, Freeform reversible embedding of suspended hydrogels (FRESH), Additive manufacturing

## Abstract

Bioprinting is a rapidly expanding technology with the ability to fabricate in vitro three-dimensional (3D) tissues in a layer-by-layer manner to ultimately produce a living tissue which physiologically resembles native in vivo tissue functionality. Unfortunately, large costs associated with commercially available bioprinters severely limit access to the technology. We investigated the potential for modifying a low-cost commercially available RepRap Prusa iteration 3 (i3) 3D printer with an open-source syringe-housed microextrusion print-head unit (universal paste extruder by Richard Horne, RichRap), that allowed for controlled deposition of cell-laden bioinks and Freeform Reversible Embedding of Suspended Hydrogels (FRESH) method-based printing.

## Graphical abstract

**Figure:**
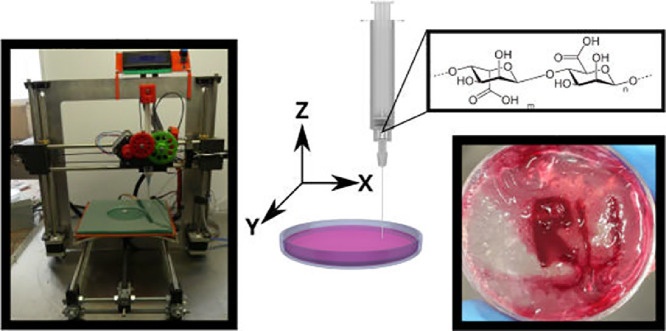

Specifications Table

**Table:** 

**Subject Area:**	Agricultural and Biological Sciences Agricultural and Biological Sciences
**More specific subject area:**	Tissue Engineering
**Protocol name:**	Assembly and firmware changes required for modifying a Prusa i3 3D printer with a RepRap paste extruder
**Reagents/tools:**	The RepRap Prusa i3 3D printer and Verbatim polylactic acid (PLA; 1.75 mm) were purchase from DIYElectronics (http://diyelectronics.co.za). Human umbilical vein endothelial cells (HUVEC; Cat No. 8000) and endothelial cell medium (ECM; Cat No. 1001) were purchased from ScienCell (Ca, USA). Adipose-derived human mesenchymal stromal/stem cells (ad-HMSC) were isolated and characterized as described (van Vollenstee et al., 2016). Trypsin/edetate disodium (EDTA) solution was purchased from Lonza Bioscience (Cat No. CC-5012). Mesenchymal stem cell medium (MSCM) purchased from ScienCell (CA, USA) (Cat No. 7501). Alginic acid sodium salt powder (Cat no. 180947), dexamethasone (Cat No. D4902), human insulin (Cat No. I3536), 3-isobutyl-1-methylxanthin (Cat No. I5879), rosiglitazone (Cat No. R2408), fluorescein diacetate (FDA; Cat No. F7378) propidium iodide (PI; Cat No. P4170), Dulbecco's Modified Eagle Medium (DMEM), minimum essential medium (MEM) eagle alpha modification (α-MEM, Cat No. M2414), Dulbecco's phosphate buffered saline (DPBS; Cat No. D8537), penicillin/streptomycin/amphotericin B (PSA) antibiotic antimycotic solution 100X (Cat No. A5955), fetal bovine serum (FBS; Cat No. F2442) and L-glutamine (Cat No. G7513) were all purchased from Sigma-Aldrich. Sterile plastic cell culture dishes were purchased from NEST. Biosafety cabinet (Class I) was purchased from Vivid Air.
***Experimental design:***	*Step-by-step description of universal paste extruder assembly and firmware modifications required for printing sodium alginate gels.*
**Trial registration:**	N/A
**Ethics:**	N/A
**Value of the Protocol:**	• Low-cost bioprinting of cell-laden alginate gels can be achieved. • The study succeeds in providing a framework upon which further modifications and refinements may improve, such that accurate and precise bioprinting applications are executed.

## Description of protocol

### Background/Introduction

Commercially available 3D bioprinters cost in the range of $4000 to >$200,000 and are typically closed-sourced and difficult to modify. Due to the high cost, few research groups have access to this technology. Here we describe the assembly and subsequent firmware modifications required for developing a RepRap Prusa i3 3D printer fitted with a syringe-housed universal paste extruder, for the printing of biological materials using the freeform reversible embedding of suspended hydrogels (FRESH) method [1], [2] at low cost. The protocol described here will contribute to the democratization of bioprinting technology and furthermore find application in biomedical education.

### RepRap Prusa i3 assembly

The RepRap Prusa i3 kit was used under the RepRap General Public License (GPL). The printer used in this study has a maximum build volume (mm) of 200 × 200 × 200 and includes dual T8 (8 mm) lead screws for improved spatial control about the Z-axis. Assembly of the printer was done as per supplier's instructions (DIYElectronics, South Africa). A similar system may be found at the RepRap.org wiki (Prusa i3, accessed at March 2020) [3].

### Universal paste extruder assembly

**Fig. 1:**
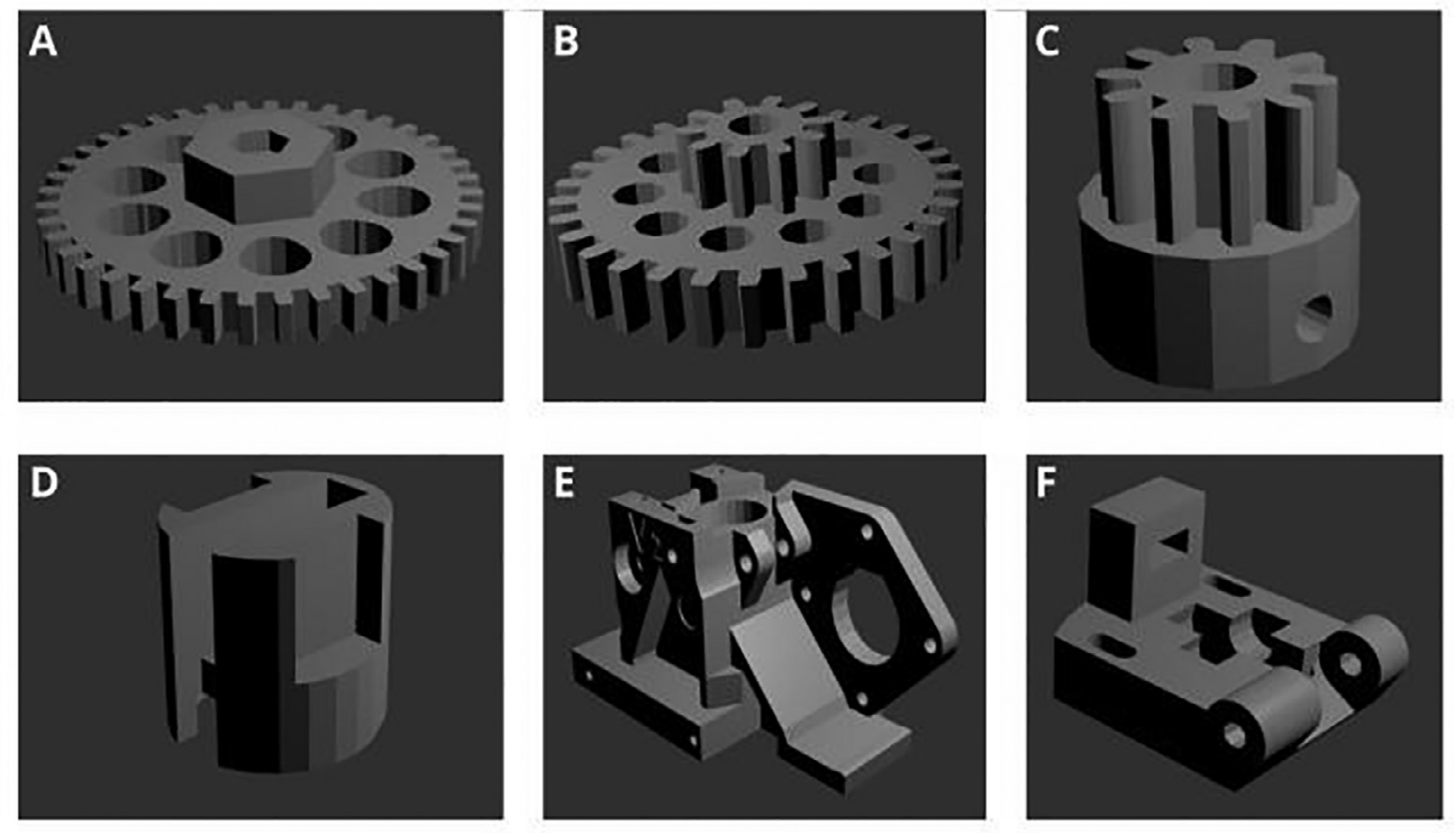
Three-dimensional (3D) printed parts required for universal paste extruder assembly. A: Large gear. B: Double gear. C: Drive gear. D: Syringe cap. E: Extruder body. F: Idler. Images were rendered in Blender (v2.78).

**Fig. 2:**
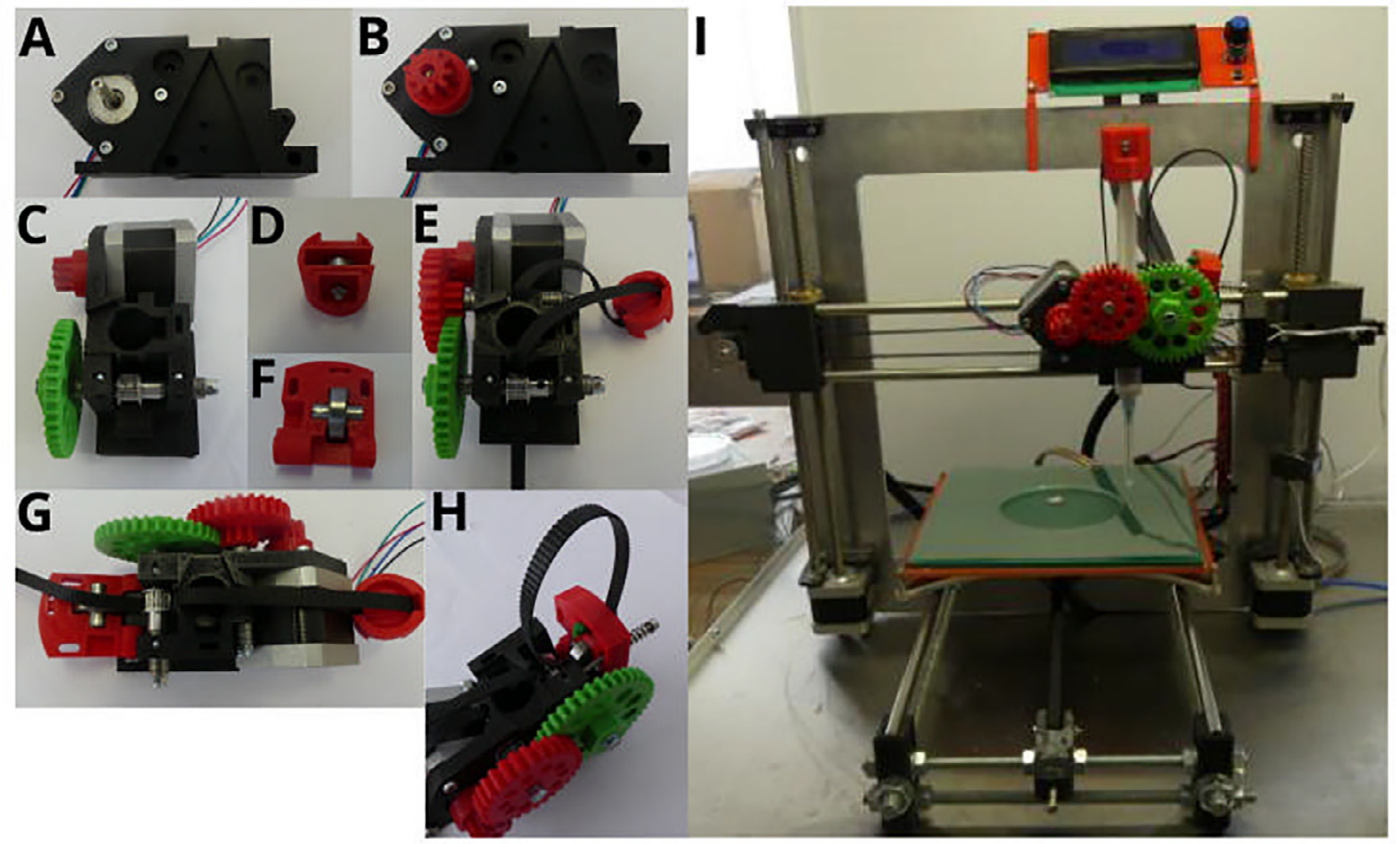
Universal paste extruder assembly process. A: Stepper motor mounting. B: Drive gear attachment. C: Large gear attachment. D: Syringe cap bearing attachment. E: Double gear attachment. F: Idler bearing attachment. G: Idler attachment. H: GT2 belt attachment, I: Modified Prusa i3 based bioprinter with mounted extruder.

The universal paste extruder 3D files were all obtained from https://www.thingiverse.com. The gears, syringe cap, as well as the idlers were downloaded from the originally uploaded universal paste extruder by user RichRap (Thingiverse #20733; licensed under the Creative Commons Attribution 3.0 Unported (CC BY 3.0) open source license). The extruder body was modified from the original such that mounting to the Prusa i3 X-carriage was made possible (Thingiverse #1125286). Printing of parts (Fig. 1) was performed using a Wanhao Duplicator i3 3D printer using a 1.75 mm Verbatim PLA filament. The universal paste extruder traditionally makes use of a 3D printed GT2 belt pulley. In the present study, an aluminum GT2 pulley (with grub screws) was favored over the printed belt pulley (as can be seen in Fig. 2C). Outlined below is a detailed explanation of how the universal paste extruder was assembled:

1. A NEMA 17 stepper motor was attached to the printed extruder body using four M3 × 10 mm screws (Fig. 2A).
2. The printed motor drive gear (small gear) was attached to the NEMA 17 stepper motor. An M3 nut was first inserted into the nut holder in the motor drive gear. The motor drive gear was then secured to the stepper motor using an M3 × 10 mm screw, through the previously inserted M3 nut (Fig. 2B).
3. The printed large gear was mounted to the extruder body by sliding an M4 × 75 mm screw through the gear, followed by eight M4 washers and a 624ZZ bearing. The M4 × 75 mm screw (with washers and bearing) was inserted through the extruder body by sliding the screw through the GT2 pulley (positioned between two M4 washers) in the middle of the extruder body. The screw was secured to the extruder body by sliding an additional 624ZZ bearing, followed by seven M4 washers and an M4 nutlock, to the end of the screw (Fig. 2C).
4. A 624ZZ bearing was inserted into the printed syringe cap by sliding an M3 × 20 mm screw through the bearing and syringe cap and securing with an M3 nut (Fig. 2D).
5. A GT2 toothed rubber timing belt was fed through the syringe cap bearing and secured to the extruder body by passing the belt through the GT2 aluminum pulley (using tweezers) and inserting the belt in the space between the NEMA 17 stepper motor and the printed extruder body (Fig. 2E).
6. The printed double gear was secured to the extruder body by sliding an M4 × 60 mm screw through the gear, followed by five M4 washers and a 624ZZ bearing. When inserting the screw through the extruder body, the GT2 belt was tightly secured to the extruder. The screw was secured to the extruder body by sliding an additional ten M4 washers, followed by an M4 nutlock (Fig. 2E).
7. A 608ZZ bearing was attached to the printed idler by inserting an M8 × 20 mm smooth rod through the 608ZZ bearing and inserting it into the idler (Fig. 2F).
8. The base of the idler was secured to the extruder body using an M4 × 35 mm screw (Fig. 2G). Two M3 nuts were inserted into the spaces provided at the top of the extruder body. The top of the idler was secured to the extruder body using an M3 × 45 mm screw (with a 15 mm spring) (Fig. 2H).
9. The entire universal paste extruder was mounted onto the Prusa i3 by screwing the printed extruder body to the X-carriage of the Prusa i3 using two M3 × 40 mm screws (Fig. 2I).
10 The NEMA 17 stepper motor cables were connected to the E0 plug on the RAMPS 1.4 board. The RAMPS 1.4 board was connected to an Arduino MEGA 2560 board and powered by an external 220 V power supply.

## Firmware

### Arduino flashing

A RAMPS/Arduino board was connected to a PC using a USB cable (USB 2.0 A to USB 2.0 C port on the Arduino board).

Firmware installation was achieved using the Arduino Integrated Development Environment (v1.6.7), which can be downloaded from: http://arduino.cc/en/Main/Software. Open-source Marlin (v1.27.6) was used for installing (i.e. flashing) the Arduino MEGA 2560 board. Marlin can be downloaded from: https://github.com/MarlinFirmware/Marlin.

Upon successful flashing and testing of the printer, the following modifications were made to the firmware to ensure movement of motors for hydrogel printing. Configurations were made in the *“Configuration.h”* tab in the Arduino Integrated Development Environment.

### Basic settings

Baud rate: Defined the speed of the serial link. The baud rate was set to *“115200”.*

Motherboard: Defined the electronics being used. *“33”* was selected for RAMPS 1.4 board.

Extruders: Defined the amount of print heads. This was set to *“1”.*

### Thermal settings

*“TEMP_SENSOR_0”* was set to *“0”,* as no extruder thermistor was used.

*“TEMP_SENSOR_BED”* was set to *“1”,* as a 100k thermistor was used for the heated print bed.

*“EXTRUDER_MINTEMP”* was set to *“0”,* as no extruder thermistor was used.

### Mechanical settings

Endstop *“INVERTING”* was set to *“false”* for all three the X, Y and Z endstops.

*“HOME_DIR”* was set to *“−1”* to set the endstops in their minimum position.

*“MIN_POS”* was set to *“0”* and *“MAX_POS”* was set to *“200”* to define the printer limits.

### Movement settings

*“NUM_AXIS”* was set to *“4”* such that all the X, Y and Z-axis, as well as an extruder motor could be controlled.

*“HOMING_FEEDRATE”* was set to *“(50×60. 50×60, 4* *× 60, 0)”* for the X, Y, Z, E-axis, respectively. This defined the speed (mm/min) the printer travelled to its home position.

*“DEFAULT_AXIS_STEPS_PER_UNIT”* was set to *“(80,80,4000,465)”.*

*“DEFAULT_RETRACT ACCELERATION”* was changed to *“1000”.*

*“DEFAULT_ACCELERATION”* was changed to *“1000”.*

JERK settings were:

*“XY 15.0”* mm/s

*“Z 0”* mm/s

*“E 7.0”* mm/s

### Printer control & slicing software

Pronterface (printrun-20150310) host interface software was used for controlling the printer X-, Y- & Z- movements, as well as the extruder motor. The software was downloaded from: https://github.com/kliment/Printrun/releases/tag/printrun-20150310.

Default Pronterface settings were used at 250 000 baud rate. Print configurations were made using Cura Software (v15.04.4).

STereoLithography (STL) files designed for printing various constructs were established using SketchUP Make (v16.0.1) software and can be downloaded from: https://www.sketchup.com/.

Preparing STL files for 3D printing (i.e. slicing) was achieved using Cura 15.4 software. Cura was downloaded from: https://ultimaker.com/en/products/cura-software. The standard configurations made in Cura are tabulated in Table 1.

**Table 1:** Standard Cura configurations used for printing.

Quality	Fill	Speed and Temperature	Support	Filament	Machine
Layer height: 1 mm	Fill density: 0%	Print speed: 2 mm/s	Support type: None	Diameter: 0.5 mm	Nozzle size: 0.5 mm
Shell thickness: 0.5 mm		Print/Bed temperature: 0 °C	Platform adhesion type: None	Flow: 5%	

### Validation of bioprinting functionality

To assess the functionality of the modified system for low-cost bioprinting, cell-laden sodium alginate gels were printed, and live/dead staining performed at one- and seven-days post printing. Additionally, non-biological freeform reversible embedding of suspended hydrogel (FRESH) printing was performed to assess printing accuracy.

The RepRap Prusa i3 was housed in a Vivid Air biosafety cabinet (class I) containing a HEPA filter and a UV sterilization lamp. sterilization of the cabinet and 3D printer was achieved by short wavelength UV radiation for one hour prior to initiating the printing process. A filtered air flow of 100 Pa was always maintained within the cabinet during the sterilization and printing processes. Aseptic technique was maintained throughout the printing experiments.

### Three-dimensional (3D) printing of cell-laden sodium alginate hydrogels

**Fig. 3:**
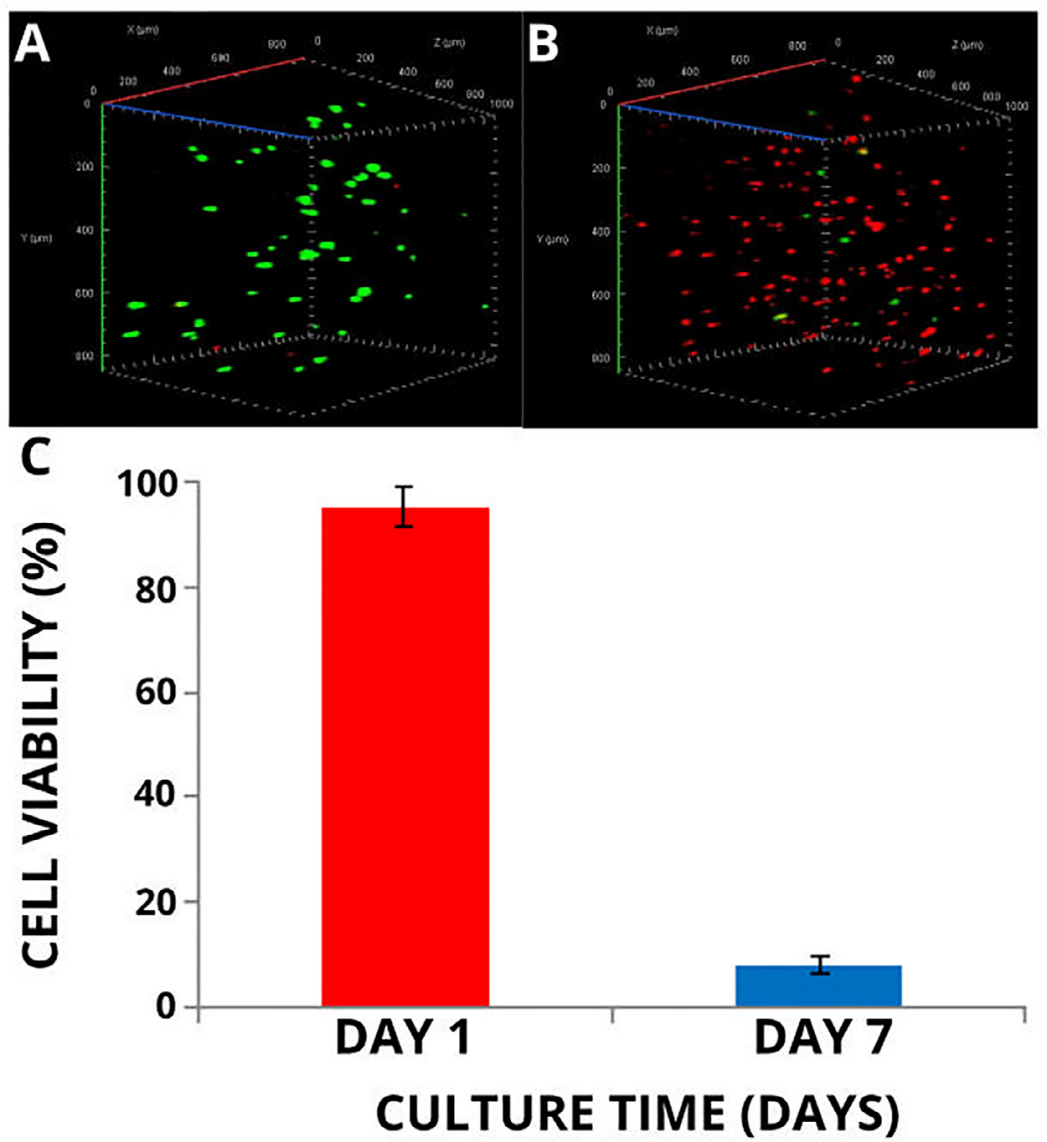
Live/dead assay of three-dimensional (3D) printed adipogenic-induced ad-HMSC/HUVEC co-culture encapsulated in sodium alginate (2% (w/v) bioink. ad-HMSC (P5) was induced for adipogenic differentiation 6 days prior to printing. A: Live/dead staining images. i) Day 1, ii) Day 7. Co-cultures were maintained in AMM:ECGM at a 1:1 ratio. Cartesian axes represented by the red (X), green (Y) and blue (Z) lines. Images were captured using a Zeiss LSM780 Laser Scanning Confocal Microscope at 100X magnification (*n* = 2). B: Cell viability calculated from duplicate live/dead images. Error bars represent standard deviation.

Three-dimensional printing of co-cultured adipose-derived human mesenchymal stromal/stem cells (ad-HMSC)/Human umbilical vein endothelial cells (HUVEC), cell-laden sodium alginate hydrogels was performed using the ReRap Prusa i3 3D printer modified with a universal paste extruder housing a 10 mL Luer-lock syringe. Preparation of the bioink involved growing ad-HMSC (P_5_) and HUVEC (P_3_) cultures to confluence in separate 75 cm^2^ cell culture flasks. Day 6 adipogenic-induced ad-HMSC co-cultured with HUVEC were used for bioprinting. Ad-HMSC were grown to confluence using α-MEM supplemented with 1% (v/v) PSA, 10% (v/v) FBS and 4 mM l-glutamine. Upon achieving confluence, ad-HMSC were induced for adipogenic differentiation for 6 days. Cells were washed with Ca^2+^ and Mg^2+^-free DPBS and fed using adipogenic induction media for 3 days. Adipogenic induction media was made up of DMEM supplemented with 1% (v/v) PSA, 10% (v/v) FBS, 1 µM dexamethasone, 10 µg/mL human insulin, 0.5 mM IBMX and 2 µM rosiglitazone. Three days after induction, media was replaced and cells were fed with adipogenic maintenance media consisting of DMEM supplemented with 1% (v/v) PSA, 10% (v/v) FBS, and 10 µg/mL human insulin only for 3 days. HUVEC were cultured in all-in-one endothelial cell medium (ECM). Upon achieving confluence, cells were resuspended in 2% (w/v) sodium alginate gel solutions made up using Ca^2+^ and Mg^2+^-free DPBS (pH 7.4) at densities of 1 × 10^6^ cells/mL per cell type. Cell-laden alginate gels were then transferred to sterile 10 mL Luer-lock syringes. Sterile 23 G syringe needles (0.34 mm needle inner diameter) were attached to the ends of syringes prior to housing within the RepRap Prusa i3. Cell-laden sodium alginate gels were printed onto sterile plastic cell culture dishes (NEST, 9.62 cm^2^), and were polymerised with sterile CaCl_2_ (30 mM) solution for 2 min. Co-cultured adipogenic-induced ad-HMSC:HUVEC constructs were maintained in 1:1 ratios of AMM:ECM cell culture media. Media was replaced with fresh AMM:ECM after 3 days. Printed cell-laden hydrogels were incubated at 37 °C, in a humidified 5% (v/v) CO_2_ atmosphere. Live/dead cell staining of 3D printed constructs was performed 1 and 7 days post-printing (Fig. 3). Cell culture medium was removed and replaced with live/dead staining solution consisting of DMEM, 1% (v/v) PSA, 19.2 µM FDA and 30 µM PI. Cells were incubated with live/dead staining solution for 4 min in the dark following removal of the staining solution. Printed constructs were then washed with Ca^2+^ and Mg^2+^-free DPBS (pH 7.4). Constructs were transferred to glass bottom culture dishes where Ca^2+^ and Mg^2+^-free DPBS (pH 7.4) was added to prevent drying of the hydrogels. Samples were analysed using a Zeiss LSM780 Confocal Laser Scanning Microscope at 100X magnification. Dead (red) cells were detected using the dsRED filter (563 nm/581 nm), whereas live (green) cells were detected using the FITC filter (495 nm/519 nm). Z-stacked fluorescence micrographs consisted of 32 slices which were captured at 30 µm intervals (960 µm Z-stack depth).

### Freeform reversible embedding of suspended hydrogels (FRESH) for printing volume fidelity

**Fig. 4:**
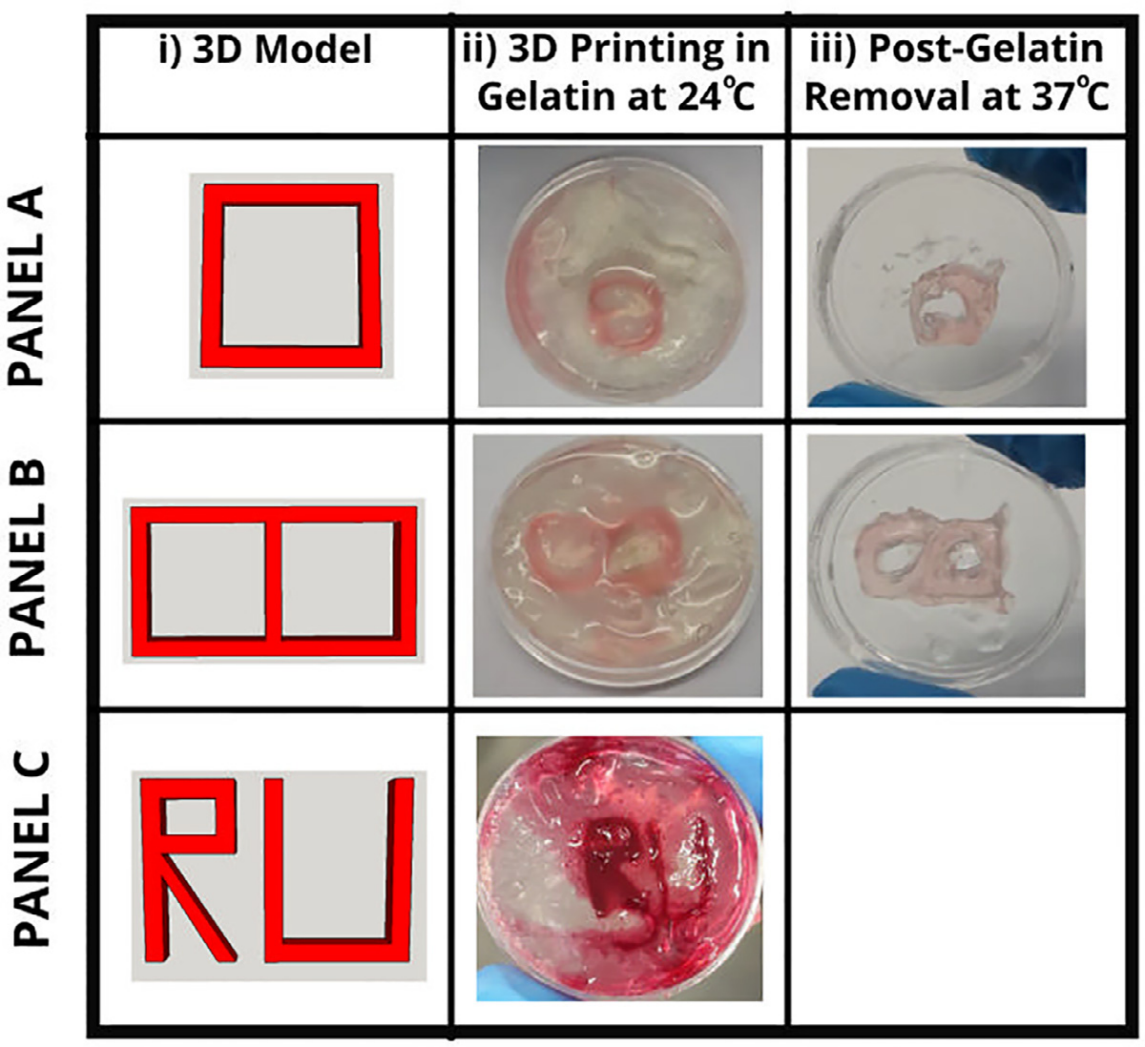
Freeform reversible embedding of suspended hydrogels (FRESH) printing through deposition of sodium alginate precursor ink within thermally reversible gelatin support bath. PANEL A: Dual-layer square construct (10 mm × 10 mm). i) Rendered STL of square, ii) Printed construct embedded within gelatin support bath (24 °C), iii) Printed alginate construct following gelatin dissolving and release of hydrogel (37 °C). PANEL B: Dual-layer grid constructs (20 mm × 10 mm). i) Rendered STL of grid, ii) Printed construct embedded within gelatin support bath (24 °C), iii) Printed alginate construct following gelatin dissolving and release of hydrogel (37 °C). PANEL C: Dual-layer RU construct (25 mm × 18 mm). i) Rendered STL of RU, ii) Printed construct embedded within gelatin support bath (24 °C). Scale bars represent 10 mm. Stereolithography images were drawn and rendered in SketchUp Make (v16.0.1).

Cell-free FRESH printing (Fig. 4) was performed using the RepRap Prusa i3 modified with a universal past extruder using methods similar to those described in Hinton et al. [2]. Gelatin slurry support baths were created by dissolving gelatin (type B) from bovine skin at 4.5% (w/v) in 30 mM CaCl_2_ solution in glass Schott bottles. Gelatin was fully dissolved by heating to 80 °C with vigorous stirring for 1 hour. The gelatin solution was then placed at 4 °C overnight for complete gelling. Next, gelatin was blended at pulse speed using a Safeway consumer-grade 1.5 L jug blender in 230 mL CaCl_2_ (30 mM) at 4 °C. Blended gelatin micro particles were transferred to 50 mL centrifuge tubes and centrifuged at 4 200 rpm for 2 min (4 °C) such that gelatin particles could be separated out of suspension. Supernatant was removed and gelatin particles were suspended back in CaCl_2_ (30 mM; 4 °C) by vortexing. The gelatin slurry was then centrifuged as before. This process was repeated until no bubbles could be seen in the supernatant, indicating that the majority of soluble gelatin had been removed. Once no air bubbles were visible, supernatant was removed, and the gelatin slurry could be used for FRESH printing.

Gelatin slurries were poured in plastic cell culture dishes (9.62 cm^2^). All FRESH printing was performed using gelatin blended for 135 s. Following FRESH printing using 2% (w/v) sodium alginate gel solutions, melting of the gelatin support bath and subsequent release of the printed alginate construct was achieved by placing plastic culture dishes on a heated plate (37 °C) for 5 min. Gelatin solution was removed by pipetting. All STL files used for FRESH printing were designed in SketchUp Make, sliced in Cura (1 mm layer height) and printed using Pronterface controller software.

The printing accuracy of FRESH printed sodium alginate (2% w/v) was determined by comparing the difference in volume of printed constructs to that of designed geometries. An STL file of a rectangular shaped construct with dimensions of (mm): 15 × 1 × 1 (length × breadth × height) (15mm^3^vol) was designed in SketchUp Make and sliced in Cura for printing of a single layer construct. The construct was designed to consist of two layers in width (0.5 mm shell thickness). A second construct with dimensions of (mm): 15 × 1 × 2 (30mm^3^vol) was designed and sliced for printing of a dual layer construct. The dual layer construct also consisted of two layers in width (0.5 mm shell thickness). The printing accuracy was calculated as the percentage overlap of printed to designed volume. Findings are highlighted in Table 2.

**Table 2:** Printing accuracy of designed construct volume to that of printed alginate. Sodium alginate (2% w/v) was FRESH printed and gelled within a CaCl_2_ (30 mM) containing gelatin support bath. Printing accuracy (%) was calculated as the percentage overlap of printed to designed volume (*n* = 3).

Layers	Designed construct volume (mm^3^)	Average printed construct volume (mm^3^)	Printing accuracy (%)
1	15	52.32 (± 4.82)	28.83 (± 2.54)
2	30	118.83 (± 26.86)	26.06 (± 5.42)

As can be seen in Fig. 4 the FRESH printing procedure resulted in deformed final printed objects relative to the designed constructs. Object shapes are further deformed by removal of the gelatin support bath and natural swelling of alginate (see in Table 2). We believe for an accurate shape analysis to be performed, X-ray microtomography (µCT) would be required for appropriate analysis.
